# 4-[(*Z*)-(*n*-Butyl­amino)(phenyl)methyl­idene]-3-methyl-1-phenyl-1*H*-pyrazol-5(4*H*)-one

**DOI:** 10.1107/S1600536812009166

**Published:** 2012-03-07

**Authors:** Hai-Wen Wang

**Affiliations:** a East China University of Science and Technology, College of Chemistry and Molecular Engineering, Mei Long Road 130, Shanghai 200237, People’s Republic of China

## Abstract

The title compound, C_21_H_23_N_3_O, exists in an enamine–keto form with the amino group involved in an intra­molecular N—H⋯O hydrogen bond. The dihedral angle between the phenyl rings is 73.59 (6)°. The five-membered ring is nearly planar, the largest deviation being 0.0004 (7) Å, and makes dihedral angles of 4.81 (6) and 69.81 (5)° wth the phenyl rings. In the crystal, pairs of weak C—H⋯O inter­actions link the mol­ecules into centrosymmetric dimers.

## Related literature

For applications of Schiff bases derived from 4-acyl­pyrazolo­nes, see: Bernardino *et al.* (2006 ▶); Zhang *et al.* (2008 ▶). For related structures, see: Zhang *et al.* (2007 ▶); Chi *et al.* (2010 ▶); Zhen & Han (2005 ▶); Wang (2010 ▶).
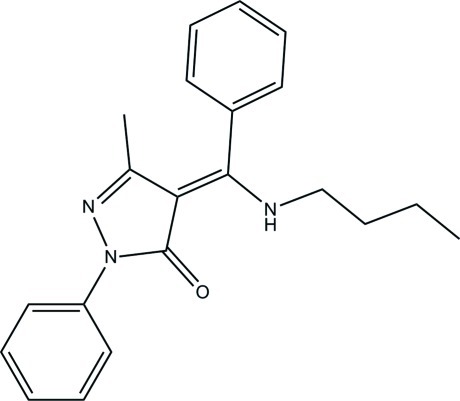


## Experimental

### 

#### Crystal data

C_21_H_23_N_3_O
*M* *_r_* = 333.42
Monoclinic, 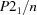

*a* = 9.5215 (9) Å
*b* = 14.7867 (14) Å
*c* = 12.8055 (12) Å
β = 100.645 (2)°
*V* = 1771.9 (3) Å^3^
*Z* = 4
Mo *K*α radiation
μ = 0.08 mm^−1^
*T* = 296 K
0.28 × 0.20 × 0.16 mm

#### Data collection

Bruker SMART 1000 CCD diffractometer
16506 measured reflections
4368 independent reflections
3362 reflections with *I* > 2σ(*I*)
*R*_int_ = 0.035

#### Refinement

*R*[*F* ^2^ > 2σ(*F* ^2^)] = 0.040
*wR*(*F* ^2^) = 0.103
*S* = 1.01
4368 reflections
232 parameters
H atoms treated by a mixture of independent and constrained refinement
Δρ_max_ = 0.30 e Å^−3^
Δρ_min_ = −0.20 e Å^−3^

### 

Data collection: *SMART* (Bruker, 2001 ▶); cell refinement: *SAINT-Plus* (Bruker, 2003 ▶); data reduction: *SAINT-Plus*; program(s) used to solve structure: *SHELXTL* (Sheldrick, 2008 ▶); program(s) used to refine structure: *SHELXTL*; molecular graphics: *SHELXTL*; software used to prepare material for publication: *SHELXTL*.

## Supplementary Material

Crystal structure: contains datablock(s) I, global. DOI: 10.1107/S1600536812009166/cv5252sup1.cif

Structure factors: contains datablock(s) I. DOI: 10.1107/S1600536812009166/cv5252Isup2.hkl

Supplementary material file. DOI: 10.1107/S1600536812009166/cv5252Isup3.cml

Additional supplementary materials:  crystallographic information; 3D view; checkCIF report

## Figures and Tables

**Table 1:** Hydrogen-bond geometry (Å, °)

*D*—H⋯*A*	*D*—H	H⋯*A*	*D*⋯*A*	*D*—H⋯*A*
N3—H3*A*⋯O1	0.921 (16)	1.873 (16)	2.6704 (14)	143.5 (14)
C13—H13⋯O1^i^	0.93	2.39	3.3175 (15)	172

Symmetry code: (i) 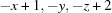.

**Fig. 1.**
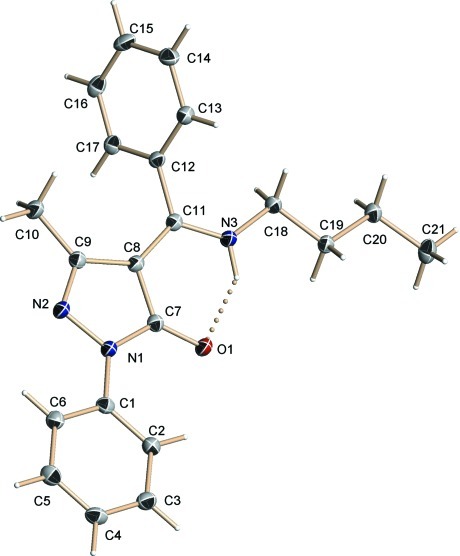
The molecular structure of (I) showing the atomic numbering and 30% probability displacement ellipsoids. Dashed line denotes hydrogen bond.

## supplementary crystallographic information

### Comment

The Schiff bases derived from 4-acylpyrazolones have attracted much attention due to their applications in pharmaceutical and agrochemical fields (*e.g.* Bernardino *et al.*, 2006; Zhang *et al.*, 2008). In order to expand this field, we now report the synthesis and structure of the title compound, (I) (Fig. 1).

In (I), the Schiff base molecule adopts an *E* geometry with respect to the C=N bond (Fig. 1). All bond lengths and angles are comparable with those found in the related compounds (Chi *et al.*, 2010; Wang *et al.*, 2010; Zhen *et al.*, 2005; Zhang *et al.*., 2007). The dihedral angle between the two phenyl rings is 73.59 (6)°. The five-membered ring of the title compound is nearly planar, with the largest deviation being 0.0004 (7)%A for atom N1. The dihedral angles between this mean plane and two benzene rings are 4.81 (6)° and 69.81 (5)%. Weak intermolecular C—H···O interactions (Table 1) link the molecules into centrosymmetric dimers.

### Experimental

A mixture of a 10 ml HPMBP (2 mmol, 0.5566 g) anhydrous ethanol solution, and a 0.2 ml n-butylamine (2 mmol, 0.1463 g) solution was refluxed for *ca* 8 h, with addition of a few drops of glacial acetic acid as a catalyst. The ethanol was removed by evaporation and the resulting green precipitate formed was filtered off, washed with cold anhydrous ethanol and dried in air. Yellow block single crystals suitable for analysis were obtained by slow evaporation of a solution in anhydrous ethanol at room temperature for a few days.

### Refinement

The H3A atom bonded to N3 was located in a difference map and isotropically refined. C-bound H atoms were placed in calculated positions, with C—H = 0.93-0.97 Å, and refined as riding, with *U*_iso_(H)=1.2-1.5 *U*_eq_ (C).

### Crystal data

**Table:**

C_21_H_23_N_3_O	*F*(000) = 712.0
*M**_r_* = 333.42	*D*_x_ = 1.250 Mg m^−^^3^
Monoclinic, *P*2_1_/*n*	Mo *K*α radiation, λ = 0.71073 Å
Hall symbol: -P 2yn	Cell parameters from 3753 reflections
*a* = 9.5215 (9) Å	θ = 2.6–28.1°
*b* = 14.7867 (14) Å	µ = 0.08 mm^−^^1^
*c* = 12.8055 (12) Å	*T* = 296 K
β = 100.645 (2)°	Block, yellow
*V* = 1771.9 (3) Å^3^	0.28 × 0.20 × 0.16 mm
*Z* = 4	

### Data collection

**Table:**

Bruker SMART 1000 CCD diffractometer	3362 reflections with *I* > 2σ(*I*)
Radiation source: fine-focus sealed tube	*R*_int_ = 0.035
Graphite monochromator	θ_max_ = 28.3°, θ_min_ = 2.6°
phi and ω scans	*h* = −12→12
16506 measured reflections	*k* = −19→19
4368 independent reflections	*l* = −17→16

### Refinement

**Table:**

Refinement on *F*^2^	Primary atom site location: structure-invariant direct methods
Least-squares matrix: full	Secondary atom site location: difference Fourier map
*R*[*F*^2^ > 2σ(*F*^2^)] = 0.040	Hydrogen site location: inferred from neighbouring sites
*wR*(*F*^2^) = 0.103	H atoms treated by a mixture of independent and constrained refinement
*S* = 1.01	*w* = 1/[σ^2^(*F*_o_^2^) + (0.0499*P*)^2^ + 0.4177*P*] where *P* = (*F*_o_^2^ + 2*F*_c_^2^)/3
4368 reflections	(Δ/σ)_max_ < 0.001
232 parameters	Δρ_max_ = 0.30 e Å^−^^3^
0 restraints	Δρ_min_ = −0.20 e Å^−^^3^

### Special details

**Table:**

Geometry. All e.s.d.'s (except the e.s.d. in the dihedral angle between two l.s. planes) are estimated using the full covariance matrix. The cell e.s.d.'s are taken into account individually in the estimation of e.s.d.'s in distances, angles and torsion angles; correlations between e.s.d.'s in cell parameters are only used when they are defined by crystal symmetry. An approximate (isotropic) treatment of cell e.s.d.'s is used for estimating e.s.d.'s involving l.s. planes.
Refinement. Refinement of *F*^2^ against ALL reflections. The weighted *R*-factor *wR* and goodness of fit *S* are based on *F*^2^, conventional *R*-factors *R* are based on *F*, with *F* set to zero for negative *F*^2^. The threshold expression of *F*^2^ > σ(*F*^2^) is used only for calculating *R*-factors(gt) *etc*. and is not relevant to the choice of reflections for refinement. *R*-factors based on *F*^2^ are statistically about twice as large as those based on *F*, and *R*- factors based on ALL data will be even larger.

### Fractional atomic coordinates and isotropic or equivalent isotropic displacement parameters (Å^2^)

**Table:**

	*x*	*y*	*z*	*U*_iso_*/*U*_eq_	
C1	0.21783 (12)	0.26068 (9)	0.88837 (9)	0.0167 (2)	
C2	0.10538 (13)	0.21696 (9)	0.92399 (9)	0.0204 (3)	
H2	0.1013	0.1541	0.9255	0.024*	
C3	−0.00037 (13)	0.26856 (9)	0.95714 (10)	0.0218 (3)	
H3	−0.0742	0.2397	0.9824	0.026*	
C4	0.00239 (13)	0.36198 (9)	0.95320 (9)	0.0218 (3)	
H4	−0.0699	0.3957	0.9744	0.026*	
C5	0.11413 (13)	0.40483 (9)	0.91726 (10)	0.0220 (3)	
H5	0.1166	0.4676	0.9144	0.026*	
C6	0.22209 (13)	0.35488 (9)	0.88561 (9)	0.0193 (3)	
H6	0.2973	0.3842	0.8626	0.023*	
C7	0.35303 (12)	0.11883 (8)	0.85902 (9)	0.0158 (2)	
C8	0.48777 (12)	0.10785 (8)	0.82441 (9)	0.0159 (2)	
C9	0.53156 (12)	0.19778 (8)	0.80232 (9)	0.0168 (2)	
C10	0.66208 (13)	0.22991 (9)	0.76371 (10)	0.0217 (3)	
H10A	0.6553	0.2136	0.6904	0.033*	
H10B	0.7453	0.2023	0.8052	0.033*	
H10C	0.6693	0.2945	0.7708	0.033*	
C11	0.55372 (12)	0.02287 (8)	0.82705 (9)	0.0152 (2)	
C12	0.69661 (12)	0.00963 (8)	0.79819 (9)	0.0153 (2)	
C13	0.81099 (13)	−0.01709 (9)	0.87666 (9)	0.0185 (3)	
H13	0.7970	−0.0279	0.9456	0.022*	
C14	0.94570 (13)	−0.02752 (9)	0.85151 (10)	0.0221 (3)	
H14	1.0223	−0.0446	0.9038	0.027*	
C15	0.96628 (13)	−0.01252 (9)	0.74855 (10)	0.0228 (3)	
H15	1.0567	−0.0195	0.7319	0.027*	
C16	0.85241 (14)	0.01285 (9)	0.67051 (10)	0.0212 (3)	
H16	0.8663	0.0221	0.6013	0.025*	
C17	0.71793 (13)	0.02451 (8)	0.69496 (9)	0.0179 (2)	
H17	0.6419	0.0423	0.6425	0.021*	
C18	0.53117 (13)	−0.14295 (8)	0.85933 (9)	0.0177 (2)	
H18A	0.6204	−0.1519	0.9088	0.021*	
H18B	0.5456	−0.1600	0.7889	0.021*	
C19	0.41494 (13)	−0.20109 (8)	0.89147 (9)	0.0172 (2)	
H19A	0.4043	−0.1852	0.9631	0.021*	
H19B	0.3250	−0.1886	0.8444	0.021*	
C20	0.44787 (13)	−0.30125 (9)	0.88723 (10)	0.0197 (3)	
H20A	0.5401	−0.3132	0.9316	0.024*	
H20B	0.4540	−0.3177	0.8149	0.024*	
C21	0.33508 (15)	−0.35963 (9)	0.92449 (11)	0.0262 (3)	
H21A	0.3317	−0.3456	0.9972	0.039*	
H21B	0.3589	−0.4223	0.9188	0.039*	
H21C	0.2435	−0.3478	0.8810	0.039*	
H3A	0.4031 (17)	−0.0341 (11)	0.8798 (12)	0.030 (4)*	
N1	0.32840 (10)	0.21097 (7)	0.85512 (8)	0.0172 (2)	
N2	0.43854 (10)	0.25859 (7)	0.82022 (8)	0.0182 (2)	
N3	0.48854 (11)	−0.04789 (7)	0.85966 (8)	0.0172 (2)	
O1	0.27412 (9)	0.06007 (6)	0.88848 (7)	0.0195 (2)	

### Atomic displacement parameters (Å^2^)

**Table:**

	*U*^11^	*U*^22^	*U*^33^	*U*^12^	*U*^13^	*U*^23^
C1	0.0156 (5)	0.0187 (6)	0.0152 (5)	0.0023 (5)	0.0014 (4)	−0.0004 (4)
C2	0.0197 (6)	0.0187 (6)	0.0231 (6)	0.0020 (5)	0.0050 (5)	0.0022 (5)
C3	0.0188 (6)	0.0255 (7)	0.0219 (6)	0.0013 (5)	0.0058 (5)	0.0014 (5)
C4	0.0203 (6)	0.0251 (7)	0.0204 (6)	0.0061 (5)	0.0048 (5)	−0.0026 (5)
C5	0.0249 (6)	0.0175 (7)	0.0234 (6)	0.0020 (5)	0.0042 (5)	−0.0032 (5)
C6	0.0189 (6)	0.0180 (6)	0.0209 (6)	−0.0008 (5)	0.0034 (4)	−0.0002 (5)
C7	0.0163 (5)	0.0158 (6)	0.0149 (5)	−0.0001 (5)	0.0019 (4)	0.0002 (4)
C8	0.0150 (5)	0.0171 (6)	0.0157 (5)	−0.0013 (4)	0.0034 (4)	0.0005 (4)
C9	0.0165 (5)	0.0171 (6)	0.0167 (5)	−0.0003 (5)	0.0026 (4)	0.0004 (4)
C10	0.0200 (6)	0.0174 (6)	0.0293 (6)	−0.0020 (5)	0.0088 (5)	0.0009 (5)
C11	0.0156 (5)	0.0171 (6)	0.0128 (5)	−0.0014 (5)	0.0019 (4)	0.0000 (4)
C12	0.0154 (5)	0.0129 (6)	0.0184 (5)	−0.0009 (4)	0.0050 (4)	−0.0011 (4)
C13	0.0184 (6)	0.0186 (6)	0.0188 (5)	−0.0011 (5)	0.0042 (4)	0.0005 (5)
C14	0.0162 (6)	0.0213 (7)	0.0282 (6)	−0.0001 (5)	0.0024 (5)	0.0004 (5)
C15	0.0170 (6)	0.0209 (7)	0.0333 (7)	−0.0030 (5)	0.0116 (5)	−0.0056 (5)
C16	0.0261 (6)	0.0189 (6)	0.0208 (6)	−0.0054 (5)	0.0106 (5)	−0.0040 (5)
C17	0.0197 (6)	0.0159 (6)	0.0183 (5)	−0.0023 (5)	0.0038 (4)	−0.0010 (5)
C18	0.0181 (5)	0.0142 (6)	0.0216 (5)	0.0010 (5)	0.0055 (4)	0.0006 (5)
C19	0.0181 (5)	0.0159 (6)	0.0182 (5)	−0.0007 (5)	0.0051 (4)	0.0012 (4)
C20	0.0218 (6)	0.0153 (6)	0.0224 (6)	−0.0006 (5)	0.0047 (5)	0.0012 (5)
C21	0.0324 (7)	0.0190 (7)	0.0283 (6)	−0.0056 (6)	0.0087 (5)	0.0008 (5)
N1	0.0159 (5)	0.0149 (5)	0.0218 (5)	0.0002 (4)	0.0064 (4)	0.0020 (4)
N2	0.0164 (5)	0.0168 (5)	0.0226 (5)	−0.0020 (4)	0.0068 (4)	0.0016 (4)
N3	0.0165 (5)	0.0142 (5)	0.0224 (5)	0.0007 (4)	0.0073 (4)	0.0009 (4)
O1	0.0180 (4)	0.0168 (5)	0.0252 (4)	−0.0018 (3)	0.0082 (3)	0.0018 (3)

### Geometric parameters (Å, º)

**Table:**

C1—C6	1.3942 (18)	C12—C13	1.3956 (16)
C1—C2	1.3975 (17)	C13—C14	1.3876 (17)
C1—N1	1.4125 (15)	C13—H13	0.9300
C2—C3	1.3906 (18)	C14—C15	1.3861 (18)
C2—H2	0.9300	C14—H14	0.9300
C3—C4	1.3827 (19)	C15—C16	1.3838 (18)
C3—H3	0.9300	C15—H15	0.9300
C4—C5	1.3879 (19)	C16—C17	1.3840 (17)
C4—H4	0.9300	C16—H16	0.9300
C5—C6	1.3858 (18)	C17—H17	0.9300
C5—H5	0.9300	C18—N3	1.4633 (16)
C6—H6	0.9300	C18—C19	1.5171 (16)
C7—O1	1.2516 (15)	C18—H18A	0.9700
C7—N1	1.3819 (16)	C18—H18B	0.9700
C7—C8	1.4418 (16)	C19—C20	1.5169 (17)
C8—C11	1.4023 (17)	C19—H19A	0.9700
C8—C9	1.4373 (17)	C19—H19B	0.9700
C9—N2	1.3117 (16)	C20—C21	1.5218 (18)
C9—C10	1.4971 (16)	C20—H20A	0.9700
C10—H10A	0.9600	C20—H20B	0.9700
C10—H10B	0.9600	C21—H21A	0.9600
C10—H10C	0.9600	C21—H21B	0.9600
C11—N3	1.3230 (15)	C21—H21C	0.9600
C11—C12	1.4872 (16)	N1—N2	1.4030 (14)
C12—C17	1.3914 (16)	N3—H3A	0.921 (16)
			
C6—C1—C2	119.87 (11)	C15—C14—H14	119.9
C6—C1—N1	119.04 (11)	C13—C14—H14	119.9
C2—C1—N1	121.09 (11)	C16—C15—C14	120.05 (11)
C3—C2—C1	119.18 (13)	C16—C15—H15	120.0
C3—C2—H2	120.4	C14—C15—H15	120.0
C1—C2—H2	120.4	C15—C16—C17	120.31 (11)
C4—C3—C2	121.15 (12)	C15—C16—H16	119.8
C4—C3—H3	119.4	C17—C16—H16	119.8
C2—C3—H3	119.4	C16—C17—C12	119.91 (11)
C3—C4—C5	119.28 (12)	C16—C17—H17	120.0
C3—C4—H4	120.4	C12—C17—H17	120.0
C5—C4—H4	120.4	N3—C18—C19	109.09 (10)
C6—C5—C4	120.60 (13)	N3—C18—H18A	109.9
C6—C5—H5	119.7	C19—C18—H18A	109.9
C4—C5—H5	119.7	N3—C18—H18B	109.9
C5—C6—C1	119.90 (12)	C19—C18—H18B	109.9
C5—C6—H6	120.0	H18A—C18—H18B	108.3
C1—C6—H6	120.0	C20—C19—C18	112.19 (10)
O1—C7—N1	126.02 (11)	C20—C19—H19A	109.2
O1—C7—C8	129.22 (12)	C18—C19—H19A	109.2
N1—C7—C8	104.75 (10)	C20—C19—H19B	109.2
C11—C8—C9	133.54 (11)	C18—C19—H19B	109.2
C11—C8—C7	120.89 (11)	H19A—C19—H19B	107.9
C9—C8—C7	105.26 (10)	C19—C20—C21	112.34 (11)
N2—C9—C8	111.72 (11)	C19—C20—H20A	109.1
N2—C9—C10	117.95 (11)	C21—C20—H20A	109.1
C8—C9—C10	130.33 (11)	C19—C20—H20B	109.1
C9—C10—H10A	109.5	C21—C20—H20B	109.1
C9—C10—H10B	109.5	H20A—C20—H20B	107.9
H10A—C10—H10B	109.5	C20—C21—H21A	109.5
C9—C10—H10C	109.5	C20—C21—H21B	109.5
H10A—C10—H10C	109.5	H21A—C21—H21B	109.5
H10B—C10—H10C	109.5	C20—C21—H21C	109.5
N3—C11—C8	118.83 (11)	H21A—C21—H21C	109.5
N3—C11—C12	118.58 (11)	H21B—C21—H21C	109.5
C8—C11—C12	122.56 (11)	C7—N1—N2	111.99 (9)
C17—C12—C13	119.83 (11)	C7—N1—C1	129.28 (10)
C17—C12—C11	121.06 (10)	N2—N1—C1	118.52 (10)
C13—C12—C11	119.10 (10)	C9—N2—N1	106.27 (10)
C14—C13—C12	119.75 (11)	C11—N3—C18	127.75 (10)
C14—C13—H13	120.1	C11—N3—H3A	113.8 (10)
C12—C13—H13	120.1	C18—N3—H3A	118.3 (10)
C15—C14—C13	120.14 (11)		
			
C6—C1—C2—C3	−0.55 (16)	C11—C12—C13—C14	−178.40 (11)
N1—C1—C2—C3	179.27 (10)	C12—C13—C14—C15	−0.77 (19)
C1—C2—C3—C4	1.43 (17)	C13—C14—C15—C16	0.0 (2)
C2—C3—C4—C5	−1.14 (18)	C14—C15—C16—C17	0.8 (2)
C3—C4—C5—C6	−0.03 (18)	C15—C16—C17—C12	−0.76 (19)
C4—C5—C6—C1	0.89 (17)	C13—C12—C17—C16	−0.06 (18)
C2—C1—C6—C5	−0.60 (17)	C11—C12—C17—C16	179.14 (11)
N1—C1—C6—C5	179.59 (10)	N3—C18—C19—C20	176.92 (9)
O1—C7—C8—C11	−4.43 (18)	C18—C19—C20—C21	177.25 (10)
N1—C7—C8—C11	174.38 (10)	O1—C7—N1—N2	178.95 (10)
O1—C7—C8—C9	−178.92 (11)	C8—C7—N1—N2	0.09 (12)
N1—C7—C8—C9	−0.11 (11)	O1—C7—N1—C1	4.41 (19)
C11—C8—C9—N2	−173.37 (12)	C8—C7—N1—C1	−174.45 (10)
C7—C8—C9—N2	0.10 (13)	C6—C1—N1—C7	174.10 (11)
C11—C8—C9—C10	6.6 (2)	C2—C1—N1—C7	−5.72 (17)
C7—C8—C9—C10	−179.93 (11)	C6—C1—N1—N2	−0.14 (15)
C9—C8—C11—N3	174.20 (12)	C2—C1—N1—N2	−179.96 (10)
C7—C8—C11—N3	1.54 (16)	C8—C9—N2—N1	−0.04 (12)
C9—C8—C11—C12	−3.62 (19)	C10—C9—N2—N1	179.98 (9)
C7—C8—C11—C12	−176.27 (10)	C7—N1—N2—C9	−0.03 (12)
N3—C11—C12—C17	116.65 (13)	C1—N1—N2—C9	175.16 (9)
C8—C11—C12—C17	−65.53 (16)	C8—C11—N3—C18	173.83 (11)
N3—C11—C12—C13	−64.14 (15)	C12—C11—N3—C18	−8.27 (17)
C8—C11—C12—C13	113.67 (13)	C19—C18—N3—C11	−173.20 (11)
C17—C12—C13—C14	0.82 (18)		

### Hydrogen-bond geometry (Å, º)

**Table:**

*D*—H···*A*	*D*—H	H···*A*	*D*···*A*	*D*—H···*A*
N3—H3*A*···O1	0.921 (16)	1.873 (16)	2.6704 (14)	143.5 (14)
C13—H13···O1^i^	0.93	2.39	3.3175 (15)	172

Symmetry code: (i) −*x*+1, −*y*, −*z*+2.
